# Inferior Dislocation of Shoulder Complicated with Undisplaced Greater Tuberosity Fracture, Rupture of the Supraspinatus Tendon, and Brachial Plexus Injury in the Elderly: Case Report and Literature Review

**DOI:** 10.1155/2020/9420184

**Published:** 2020-05-30

**Authors:** Angelo V. Vasiliadis, Christos Kalitsis, Theofanis Kantas, George Biniaris

**Affiliations:** ^1^Department of Orthopaedic Surgery, General Hospital of Katerini, Katerini, Greece; ^2^School of Medicine, Aristotle University of Thessaloniki, Thessaloniki, Greece

## Abstract

Inferior dislocation of the shoulder, also called luxatio erecta, is a rare form of the otherwise common shoulder dislocation. It appears in less than 0.5% of all shoulder dislocations. An awareness of associated potential axillary artery injury, brachial plexus complications, and rotator cuff tears is important in this rare entity and should be excluded with a high index of suspicion. In our case report, we have an 83-year-old female who inferiorly dislocated her dominant shoulder with brachial plexus injury and musculotendinous injury, which was caused by an accidental fall. The dislocation was manually reduced at the emergency department. After 18 months of conservative treatment with physical therapy, the range of motion and muscle strength of the shoulder recovered to a satisfactory mobile level according to the patient's demands.

## 1. Introduction

The shoulder joint is a highly mobile, but inherently unstable one and involves articulation between the head of the humerus and the glenoid fossa of the scapula, which enables a wide range of movements including forward flexion, abduction, adduction, external/internal rotation, and 360° circumduction [1]. Shoulder dislocation is a common dislocation found in 45% of all large joint dislocations treated in an Emergency Department (ED). Inferior shoulder dislocation, also known as “*luxatio erecta*”, is a relatively rare form of glenohumeral dislocations in the ED, found in only 0.5% of all shoulder dislocations [1, 2]. Typically, the patient presents with the arm locked in an upright position [3]. Inferior shoulder dislocation is frequently associated with injuries to the static and/or dynamic stabilizers, fracture of the greater tuberosity, and can potentially be complicated with neurovascular deficit. Immediate close reduction is required to avoid neurovascular complications. A careful neurovascular examination is mandatory, both before and after closed reduction [1]. The aim of this case report is to describe an inferior shoulder dislocation with peripheral nerve injury and also give an overview regarding the neural deficit of inferior shoulder dislocation which is described in the literature.

## 2. Case Report

An 83-year-old woman came to the emergency department with a complaint of shoulder pain and incapacity to lower her arm after an accidental fall from the last step of her home staircase. According to her own report, she had put her right arm outstretched above the level of her head, in the opposite wall, causing hyperflexion of the shoulder, in order to avoid the impact of a head injury. At that moment, she experienced numbness and shock-like electrical sensations in the fingers of the ipsilateral hand. In addition, there was a clear medical history without previous shoulder dislocation or contributing pathologies.

**Figure 1:**
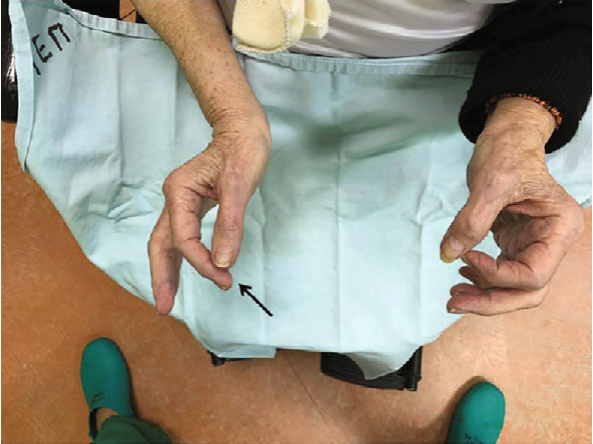
Injury of the anterior interosseous nerve with difficulty in performing the “OK” sign (black arrow).

On physical examination, her dominant arm was abducted at the shoulder and flexed at the elbow. The patient could not adduct the arm to less than 80°. Examination revealed loss of contour of shoulder and presence of the head of humerus palpable in the axilla. Vascular function was not compromised in the affected extremity, as verified through the presence of radial pulse. However, she presented a deficit attempting the “OK” sign and a positive digit abduction/adduction test, which characterizes an injury to the anterior interosseous nerve (branch of the median nerve) (Figure 1) and ulnar nerve, respectively.

**Figure 2:**
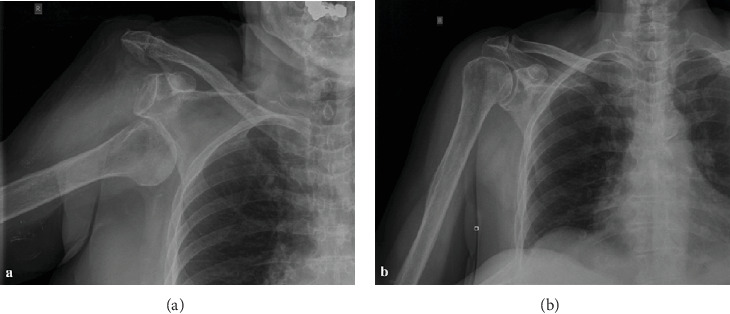
Anteroposterior (AP) radiographic view of the right shoulder shows a humeral head out of the glenoid fossa lying inferior to the glenoid rim (a). Postreduction AP radiographic view shows congruent reduction (b).

Radiographic examination confirmed the clinical diagnosis of a complete inferior dislocation of the humeral head lying beneath the glenoid cavity, without any accompanying fracture (Figure 2(a)). Immediate reduction was done with intravenous analgesia-sedation by in-line traction-abduction and gently pushing the humeral head superiorly, while countertraction was provided by an assistant. An audible pop was heard when the humeral head was reduced (Figure 2(b)). The delay between the time of injury and reduction was 2 hours. After successfully closed reduction, the right shoulder was immobilized using an arm sling. Following reduction, the deficit in attempting the “OK” sign and digit abduction/adduction test remained. The patient was discharged under instructions to maintain the immobilization (elbow to body) for three weeks to allow soft tissue healing.

**Figure 3:**
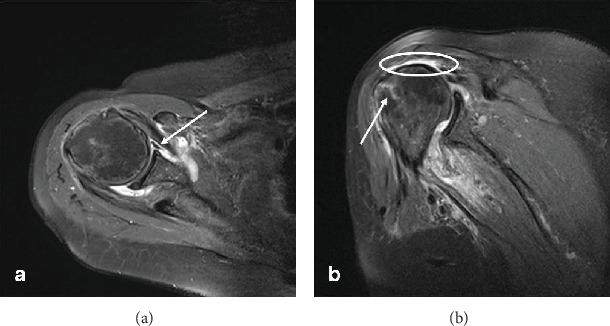
A transverse (a) T2-weighted MRI view shows a Bankart lesion (white arrow) of the right shoulder. A coronal (b) T2-weighted MRI view shows a bone marrow contusion line associated with a contusion-crack fracture without displacement of the greater tuberosity of the humerus (white arrow) and a complete rupture of the supraspinatus tendon (white circle).

A magnetic resonance imaging (MRI) of the shoulder was carried out ten days later in order to evaluate soft-tissue structures, such as the rotator cuff and labrum, and occult skeletal pathology. The MRI revealed a Bankart lesion in the anteroinferior aspect of the glenoid labrum of the shoulder. A bone marrow contusion line in the greater tuberosity of the humerus showed a contusion-crank fracture without displacement. A complete rupture of the supraspinatus tendon was also revealed (Figure 3). Although the patient was advised to treat the rotator cuff rupture surgically, she chose not to do so. Further evaluation by an electromyography study revealed the destruction of the myelin sheath of the median nerves bilaterally (carpal tunnel syndrome). In addition, the altered dynamics of the right median and ulnar nerves could be related to neurapraxia due to the nerve compression.

After three weeks, physical therapy was proposed with gentle pendulum movements of the injured upper limb. Passive range of motion and isometric deltoid exercises was proposed during the 4^th^ and 6^th^ weeks, respectively. After the 8^th^ week, active/strengthening exercises were proposed. After 6 months of the reduction, the patient had a mild weakness in the little finger, with difficulty to abduct her arm above the head (over 100°). Shoulder function was assessed, and the Constant score was 69 out of 100 points. At 18 months, the patient regained a nearly full active range of motion in the shoulder and had no instability toward elevation or rotation. At the present time point, she is satisfied with her function and she is independent and able to perform activities of daily living. The Constant score was 81 out of 100 points.

## 3. Discussion

The current case report highlights the need for awareness of the potential nerve damage following inferior shoulder dislocation and also highlights what it is known from the literature. Inferior shoulder dislocation is a rare injury and has been determined in the literature comprising only 0.5% of all shoulder dislocations with no age predilection, but with male predominance [4]. Two mechanisms of injury have been described for inferior shoulder dislocation. First, an indirect mechanism of the injury involves a sudden and forceful hyperabduction to an already abducted arm [5, 6]. The second mechanism of injury involves direct axial loading on a fully abducted arm with the elbow extended and forearm pronated [7–9].  Table 1 shows the etiology and mechanisms of injury after a review of the literature. The most common etiologies of such injury are falls, followed by sports-related injuries, motorbike, and motor vehicle accidents (Table 1).

Clinically, the patient will present with an abducted extremity, which will be unable to lower. The elbow is flexed and the forearm pronated. The hand is often resting on, or next to, the head [10, 11]. The average reported abduction of the humerus is 120° (80°-170°) on clinical assessment at presentation [3, 8, 11, 16, 20]. Closed reduction is typically performed either by manual manipulation of “traction-countertraction” method, as described by Freundlich [12], or by a two-step technique, as described by Nho et al. [13]. Several complications are reported for inferior shoulder dislocation. Bone fractures, such as great tuberosity, inferior glenoid fossa, acromion, scapular spine, coracoid process, and/or soft tissue injuries, such as avulsed shoulder capsules, rotator cuffs tears, or ruptures of the adjacent muscles (supraspinatus, infraspinatus, teres minor, sunscapularis) are likely with inferior shoulder dislocations [14, 15]. Other complications are neurovascular compromise that may affect the brachial plexus (most commonly injury to the axillary nerve) and even an axillary vein causing thrombosis [4, 14, 16].

Neurological injury after inferior shoulder dislocation was noted in 26 patients in previous reports (Table 2). In 19 patients, neurological symptoms were resolved 12 months after injury. Six patients experienced a persistent neurological deficit. No follow-up information was available for one patient who initially had radial paresthesia. Brachial plexus injuries are a well-recognized complication of inferior glenohumeral dislocation [1, 3, 17]. This is probably due to the final position of the humeral head in the axilla after the dislocation [13, 18]. It seems that the axillary nerve was probably damaged more frequently (Table 2) [1, 4, 7, 11, 18] due to its anatomic position close to the anterior aspect of the glenohumeral joint and its course around the neck of the humerus [1, 18]. The nerve path makes it liable to be stretched or/and compressed due to the humeral head when the upper limb is forced in hyperabduction of proximal humerus accompanying by direct axial loading [19, 20]. In our case report, the patient had persistent mild neurological symptoms in the distribution of the ulnar nerves after 18 months of shoulder reduction, without affecting the level of activities of daily living.

## 4. Conclusion

In conclusion, inferior shoulder dislocation requires careful clinical and radiographic evaluation and a high index of suspicion for associated injuries (bone, soft tissue, and neurovascular injuries). These injuries occur frequently and can be significantly associated with higher energy trauma. The prognosis is directly influenced by the early diagnosis and the successful reduction of shoulder dislocation. Although some authors have recommended immediate reduction followed by surgical repair of the associated injuries (such as rotator cuff), our patient achieved satisfactory functional recovery, according to the patient's demands, after immediate reduction and conservative treatment with physical therapy.

## Figures and Tables

**Table 1:** Etiology and mechanism of injuries (literature review and present study).

Study	Gender (M/F)	Age (Y)	Side, U/B (° blocked)	Etiology of injury	Mechanism of injury
Present study, 2020	F	83	U (80°)	Fall from height	Direct
Kaziz et al., 2018	F	23	U	Sport related	Direct
Yao et al., 2018	M	40	U (170°)	Fall from height	Indirect
Demirtas et al., 2017	F	80	U (130°)	Pedestrian (vs. vehicle)	Direct
	M	34	U (120°)	Fall	Direct
Fox and Martin, 2016	M	58	B	Fall from height	Indirect
Owen et al., 2016	M	30	U (170°)	MBA	Direct
Acosta et al., 2015	M	43	B (110°)	Fall from height	Direct
Cift et al., 2015	M	65	U	MVA	N/R
Petty et al., 2014	M	68	B	Sport related (treadmill)	Indirect
Ellanti et al., 2013	M	19	B	MVA	N/R
Imerci et al., 2013	M	50	U	MBA	N/R
	F	51	U	Fall	N/R
Groh et al., 2010	M	17	U	MBA	N/R
	M	17	U	Sport related (bull riding)	N/R
	M	60	U	MBA	N/R
	M	27	U	MBA	N/R
	M	17	U	Sport related (basketball)	N/R
	M	40	B	Fall from height	N/R
	F	18	U	MVA	N/R
Camarda et al., 2009	F	70	U	Fall	Indirect
Sharma and Denolf, 2004	M	69	U	Fall	Direct
Kumar et al., 2001	M	58	B (100°)	Fall	N/R
Grate, 2000	M	20	U (110°)	Sport related (wrestling)	Direct
Davids and Talbott, 1990	M	29	U	Sport related (roller skate)	Indirect
Rae and Sylvester, 1988	F	34	U	Sport related (tennis)	Indirect

Abbreviations: M: male; F: female; Y: years; U: unilateral; B: bilateral; MBA: motorbike accident; MVA: motor vehicle accident; N/R: not reported.

**Table 2:** A summary of the literature review and the present study, on the nerve injury and associated injuries of inferior shoulder dislocation.

Study	Gender (M/F)	Age (Y)	Side, U/B (° blocked)	Nerve injury	Nerve deficit (postreduction)	Associated injuries	F-U (months)
Present study, 2020	F	83	U (80°)	Medial/ulnar nerve	Ulnar nerve	GTF, RCT	18
Kaziz et al., 2018	F	23	U	Axillary nerve	None	GTF	10
Yao et al., 2018	M	40	U (150°)	Axillary nerve	None	GTF	8
Demirtas et al., 2017	F	80	U (130°)	Radial/median/ulnar nerve	None	None	26
	M	34	U (120°)	Radial nerve	None	GTF	28
Fox and Martin, 2016	M	58	B	Axillary nerve (sensory)	None	GTF, RCT, OEF	6
Owen et al., 2016	M	30	U (170°)	Brachial plexus (3 cords)	Median/ulna nerves (sensory)	GTF, RCT	24
Acosta et al., 2015	M	43	B (110°)	Radial nerve	Radial nerve (motor)	GTF	60
Cift et al., 2015	M	65	U	Radial paresthesia	None	None	22
Petty et al., 2014	M	68	B	Numbness C6-C8 (hand)	N/R	None	N/R
Ellanti et al., 2013	M	19	B	Paraesthesia C6-C7, radial nerve	None	GTF	6
Imerci et al., 2013	M	50	U	Axillary nerve	None	None	27
	F	51	U	Brachial plexus	None	None	25
Groh et al., 2010	M	17	U	Radial/median/ulnar nerve	None	None	12
	M	17	U	Ulnar nerve	None	None	264
	M	60	U	Axillary nerve	None	GTF	24
	M	27	U	Axillary/radial nerve	None	RCT	180
	M	17	U	Axillary nerve	None	None	36
	M	40	B	Axillary nerve	Axillary nerve (paresis)	None	40
	F	18	U	Axillary/radial nerve	None	None	32
Camarda et al., 2009	F	70	U	Paraesthesia C5-T1	None	None	12
Sharma and Denolf, 2004	M	69	U	Paraesthesia SCNF	None	GTF	6
Kumar et al., 2001	M	58	B (100°)	Paraesthesia C5-T1, brachial plexus	Musculocutaneous/radial/axillary (motor)	None	6
Grate, 2000	M	20	U (110°)	Tingling sensation (fingers)	None	GTF	N/R
Davids and Talbott, 1990	M	29	U	Numbness C5-T1 (arm)	None	HSL	1
Rae and Sylvester, 1988	F	34	U	Brachial plexus (3 cords)	Slight disability (hand)	None	12

Abbreviations: M: male; F: female; Y: years; U: unilateral; B: bilateral; MBA: motorbike accident; MVA: motor vehicle accident; N/R: not reported; SCNF: superficial cutaneous nerve of the forearm; GTF: great tuberosity fracture; RCT: rotator cuff tear; OEF: open elbow fracture; HSL: Hill-Sachs lesion; F-U: follow-up.
